# Anxiolytic and Sedative Properties of Melatonin Premedication in Pediatrics Undergoing Elective Cardiac Catheterization: A Randomized Placebo Study

**DOI:** 10.7759/cureus.56543

**Published:** 2024-03-20

**Authors:** Hussein A Hussein, Mohamed Kahloul, Majid F Alhamaidah, Hussein J Alkhfaji

**Affiliations:** 1 Department of Anesthesia and Intensive Care, Faculty of Medicine, University of Sousse, Sousse, TUN; 2 Department of Anesthesia and Intensive Care, Faculty of Medicine, Sahloul Teaching Hospital, University of Sousse, Sousse, TUN; 3 Department of Anesthesiology, College of Health and Medical Technology, Al-Ayen Iraqi University, Thi-Qar, IRQ

**Keywords:** ease of venepuncture, sedation, preoperative anxiety, pediatric, mask acceptance, premedication, oral melatonin

## Abstract

Background: Preoperative anxiety in children has been linked to various postoperative consequences, such as postoperative regressive behavioral issues, extended distress during the recovery period, eating disorders, and bedwetting. The current study aimed to investigate the efficacy of low-dose oral melatonin in alleviating preoperative anxiety among children in the Iraqi population.

Study design: A randomized, double-blinded comparative study was undertaken, involving children aged four to 14 years scheduled for elective cardiac catheterization under general anesthesia. The study comprised a total of 80 children. The involved individuals were randomly assigned to two groups, each with 40 subjects. Group A received 0.5 mg/kg melatonin as premedication, while Group B received a placebo.

Results: The two groups demonstrated similarity in mean age, weight, cardiac disease, and gender distribution. Statistically significant reductions in anxiety scores were observed in the melatonin group compared to the placebo group. Particularly, children administered 0.5 mg/kg melatonin exhibited the most substantial anxiolysis and venipuncture compliance (*P* < 0.05). Additionally, children who were premedicated with melatonin experienced decreased cognition, maximum sedation, successful parental separation, and psychomotor impairment (*P* < 0.05).

Conclusions: Melatonin demonstrated an effective sedation level without significant side effects, making it a preferred choice due to its efficacy, safety, current availability, and cost-effectiveness compared to other anesthetic agents used in premedication procedures.

## Introduction

Indeed, approximately 65% of children undergoing surgery and anesthesia experience significant anxiety and stress, particularly in the preoperative holding area. This situation often extends to the parents, exacerbating the child's response before surgery. The severity of child anxiety has been identified as a strong predictor for the occurrence of hallucinations during the recovery period and the development of new-onset adverse behavioral changes, such as nightmares and enuresis [1]. Overall, if patients' preoperative anxiety is not effectively managed or mitigated through premedication, it can impact surgical outcomes, including postoperative pain, analgesic needs, and length of hospital stay. Numerous systemic physiological changes may accompany anxiety, such as elevated respiratory rate, increased blood pressure, and heightened heart rate, potentially leading to the development of arrhythmias. These physiological responses can present challenges for anesthesiologists when selecting the most suitable anesthesia method or medications for induction and maintenance [2]. In the early 1980s, midazolam emerged as a superior oral premedication agent for children in the preoperative period, effectively reducing anxiety. Its swift acceptance as the preferred primer before anesthesia induction was largely due to its rapid absorption upon oral administration, versatility in route administration, and lower incidence of nausea compared to other benzodiazepines. These characteristics collectively favored midazolam as the premedication of choice in pediatric preoperative settings [3]. Although midazolam offers notable advantages when used as a premedication agent before surgical interventions, particularly for children, it also presents certain drawbacks that could impact the outcomes of the surgical procedure and the anesthesia plan [4]. Common drawbacks of midazolam include paradoxical reactions, interactions with opioids, delayed awakening during the recovery period, and inconsistent bioavailability. These issues are known to vary depending on the patient's age [3,5]. Given the drawbacks associated with the use of midazolam, the introduction of a viable alternative may be well received. Melatonin, recognized for its effectiveness as a hypnotic medication, has been shown to impact both the onset and maintenance of sleep [6]. Acting as a natural sleep-inducing agent, melatonin's effects are mediated by MT1 and MT2 receptors, as well as a physiological mechanism that is not yet fully elucidated, which governs its analgesic properties [7]. The present research sought to examine the effectiveness of oral melatonin in reducing preoperative anxiety among children in the Iraqi population compared to placebo premedication.

## Materials and methods

Study methodology

This randomized, controlled, double-blind study was conducted at the Nasiriya Heart Center in the city of Nasiriya after obtaining approval from the Ethics Committee in the Dhi Qar Health Department, affiliated with the Iraqi Ministry of Health, on September 21, 2021, approval number 36/2021. The study was registered on the Clinicaltrail.gov PRS with code NCT06031961. The study was conducted, and samples were collected between February 2022 and April 2023.

Samples of the study

The study was conducted on 80 children undergoing cardiac catheterization. All children aged between four and 12 years within the American Society of Anesthesiologists (ASA) classifications II and III, who were diagnosed with congenital atrial septal defects (primary or secondary) or ventricular septal defects and undergoing scheduled therapeutic catheter interventions in the specified location and period, were included in this study.

The exclusion criteria include the following: ASA more than III, allergy to the study drug, any contraindications for the study drug, preoperative vomiting, gastrointestinal disorders, mental and neurological diseases, multi-congenital deformities in the heart, and disapproval or dissatisfaction with the child's parents.

The participants were randomly assigned to receive premedication with melatonin or placebo 120 min before the induction of general anesthesia. The parents of participating children received thorough information about the study's objectives before signing the informed consent forms. Subsequently, the children were categorized into two groups, namely the melatonin group and the placebo group, following the acquisition of written informed consent from their parents. The sample size was determined based on previous studies in pediatric anesthesia to provide a power of 90% with a confidence interval of 99%.

Randomization and premedication

The involved children were randomly divided using the closed envelope method into two groups: the melatonin group (M group) and the Placebo (P group) group. In the M group (*n *= 40), patients received oral melatonin (0.5 mg/kg) on the morning of intervention; the maximum dose was 20 mg. In the P group (*n *= 40), patients received an identical-looking placebo 120 minutes before induction of anesthesia. Premeditations of this study were prepared by the investigator anesthesiologist and administered by another observer, and the attending anesthesiologists were blinded to the given drug.

Anesthesia procedure

The initiation of anesthesia involved the placement of an intravenous line. General anesthesia was induced by administering a sevoflurane inhalation agent (8%) through a face mask, coupled with a 100% oxygen mixture. Intravenous anesthetic agents, including fentanyl (1.5 μg/kg via slow intravenous injection), propofol (2 mg/kg), and rocuronium (0.9 mg/kg), were subsequently administered to induce neuromuscular blockade and facilitate intubation. Throughout the procedure, patients were mechanically ventilated using pressure-controlled ventilation with a 50% oxygen mixture. Anesthesia levels were adjusted to maintain stable blood pressure, heart rate, and respiratory rate within 20% of their baseline values. Standard monitoring, such as continuous electrocardiogram, noninvasive blood pressure measurement, and pulse oximetry, was implemented. After the intervention, the administration of anesthetic gases was gradually reduced to 0%, replaced by 100% oxygen at a flow rate of at least 4 L/minute. Once consciousness was regained, the endotracheal tube was removed, and the child was carefully transferred to the post-anesthetic care unit (PACU) under the supervision of an anesthesiologist. Vital signs were monitored in the PACU until the patient was deemed stable and ready for discharge to the ward.

Data collection and analysis

Child anxiety levels were assessed by a blinded observer before giving premedication (the baseline reading), 30 minutes after premedication, and at the time of separation from parents using a four-point scale: 1 = Crying; 2 = Anxious; 3 = Calm but not cooperative; and 4 = Calm, cooperative, or asleep.

The sedation level was assessed by a blinded observer before giving premedication (the baseline reading), 30 minutes after premedication, and at the time of separation from parents using a four-point scale: 1 = Alert, 2 = Awake, 3 = Drowsy, and 4 = Asleep. 

The acceptance of the mask or response to gaseous induction was assessed at the time of induction starting using the mask acceptance score, which is a five-point scale: 1 = Combative crying; 2 = Moderate fear of mask; 3 = Cooperative with assurance; 4 = Calm, cooperative; and 5 = Asleep. Scores 1 and 2 were considered unsatisfactory, and scores 3, 4, and 5 were considered satisfactory acceptance of the mask.

The ease of venipuncture was evaluated at the time of cannulation using a four-point scoring system [8]: 1 = Crying; 2 = Yelling; 3 = Limb moving; and 4 = No reaction. A score of 3 or 4 was considered as an acceptable attitude.

Statistical analysis was performed using IBM SPSS Statistics for Windows, Version 25.0 (IBM Corp., Armonk, NY), with descriptive statistics, student's t-test, mean, standard deviation (±SD), and chi-square test utilized, as appropriate, for data presentation and analysis.

## Results

Both study groups were comparable regarding the following factors: age (*P *= 0.55), gender (*P *= 1), weight (*P *= 0.57), ASA status (*P *= 0.812), and underlying disease (Table 1).

**Table 1:** Comparison between groups regarding demographic data. SD, standard deviation; ASA, American Society of Anesthesiology; ꭓ2, chi-square

Demographic data	Melatonin group (*n* = 40)	Placebo group (*n* = 40)	Test	*P*-value
Age (years)				
Mean ± SD	6.68 ± 2.12	6.95 ± 2.06	*t* = 0.588	0.558
Sex, *n* (%)				
Male	30 (75)	30 (75)	*ꭓ*^2 ^= 0.0	1.000
Female	10 (25)	10 (25)		
Weight (kg)				
Mean ± SD	22.85 ± 5.70	23.63 ± 6.46	*t* = 0.569	0.571
ASA classification, *n* (%)				
II	32 (80)	33 (82.5)	*ꭓ*^2^ = 1.033	0.812
III	8 (20)	7 (17.5)		

The anxiety and sedation levels in the M group were significantly lower than those in the P group.

Regarding the anxiety and sedation levels, before the administration of the premedication, in both groups, anxiety and sedation were comparable. After 30 minutes of taking melatonin, the anxiety score was significantly lower in the M group than in the P group (*P* = 0.001). According to our results, at this time point, most of the pediatrics were calm, cooperative, or asleep in the M group, while in the P group, we found most individuals were anxious and some of them were calm, but not cooperative.

The sedation levels in the M group were significantly lower than those in the P group (*P* = 0.001). Most of the included children in the M group were scored under 3 (in a drowsy situation), with some scoring under 4 (sleepy), while in the P group, most individuals were scored as alert or awake only (Table 2).

**Table 2:** Comparison between groups regarding sedation and anxiety levels. ^*^P-value representing the mean sedation level. M group, melatonin group; P group, placebo group

Groups	Before premedication		30 minutes after premedication		At separation from parents	
	Anxiety	Sedation	Anxiety	Sedation	Anxiety	Sedation
M group	1.80 ± 0.56	1.74 ± 0.56	3.32 ± 0.73	2.97 ± 0.77	2.42 ± 0.93	2.97 ± 0.77
P group	1.67 ± 0.57	1.65 ± 0.68	1.95 ± 0.75	1.55 ± 0.68	2.20 ± 0.56	2.30 ± 0.52
*P*-value	0.661	0.762	0.001*	0.001*	0.003*	0.001*

Regarding the anxiety score at separation time from parents, the results showed a significant difference between the groups (*P* = 0.003). The mean score of anxiety at this time point indicated that most children were calm but not cooperative in the M group, while few children were scored as anxious when separating from their parents. The results of sedation at separation time did not differ significantly from those at 30 minutes after premedication (*P *= 0.001^*^). The asterisk (*) indicates that the *P*-value remained consistent across both time points (0.001), namely, 30 minutes after premedication and at separation time.

These results imply that premedication with melatonin proved to be effective in inducing substantial anxiolytic and sedative effects in children before surgery (Table 2).

The child's reaction to anesthesia mask application was evaluated depending on five-point scores. The results showed there was a significant effect for melatonin compared to placebo. In the M group, 4 (10%) children were combative and cried, 4 (10%) had a moderate fear of the mask, 17 (42.5%) were cooperative with assurance, 15 (37.5%) were calm and cooperative, and there was no sleepy individual at that time. While in the placebo group, 11 (27.5%) children were combative and cried, 17 (42.5%) had a moderate fear of the mask, 11 (27.5%) were cooperative with assurance, and 1 (2.5%) were calm and cooperative. This means that the percentage of children who were calm and cooperative with the mask was significantly higher in the M group than in the P group (*P* = 0.001) (Table 3).

**Table 3:** Comparison between groups regarding mask acceptance. ^*^P-value representing the mean sedation level. ꭓ^2^, chi-square

	Melatonin group (*N* = 40)		Placebo group (*N* = 40)		Test	*P*-value
	n	%	n	%		
Mask acceptance						
Combative crying	4	10.0	11	27.5	*ꭓ*^2 ^= 24.850	0.001*
Moderate fear of mask	4	10.0	17	42.5		
Cooperative with assurance	17	42.5	11	27.5		
Calm and cooperative	15	37.5	1	2.5		
Asleep	0	0.0	0	0.0		

Regarding the ease of venipuncture, peripheral cannulation was performed when the child was placed on the special bed of the catheterization room. The reaction of a child to the cannulation procedure was directly evaluated using a four-point score. There was a statistically significant difference in the ease of venipuncture score between the two groups. The children who received melatonin were significantly less likely to cry, yell, or move their limbs during the venipuncture than children who received a placebo. Six (15%) children in the M group did not show any reaction to cannulation and more than 45% showed a mild reaction through simple limb movement during cannulation. However, in the P group, more than 30% were crying and 16 (40%) were yelling during cannulation (Table 4).

**Table 4:** Comparison between groups regarding the ease of venepuncture score. ^*^P-value representing the mean sedation level. ꭓ^2^, chi-square

	Melatonin group (*N* = 40)		Placebo group (*N* = 40)		Test	*P*-value
	n	%	n	%		
The ease of venepuncture score						
Crying	2	5.0	13	32.5	*ꭓ*^2 ^= 16.817	0.001*
Yelling	13	32.5	16	40.0		
Limb moving	19	47.5	11	27.5		
No reaction	6	15.0	0	0.0		

## Discussion

Based on the data from our study, there were no significant differences observed in the demographic information of the individuals involved. Regarding anxiety and sedation levels, we found that premedication with 0.5 mg/kg of oral melatonin significantly reduced anxiety levels in children, particularly during separation from their parents. Additionally, oral melatonin as a premedication agent provided significant sedation for easing separation from parents and facilitating the induction of anesthesia.

Our results indicated that children's reactions to the anesthesia mask were significantly lower in the M group compared to the P group.

In terms of the ease of venipuncture, our findings showed that children premedicated with oral melatonin exhibited fewer reactions, such as crying, yelling, or limb movements, during the venipuncture procedure, whereas the majority of children in the P group experienced crying and yelling during cannulation.

Anesthesia and surgery create a great psychological stress in most of the patients [9]. The premedication properties of oral melatonin have been investigated in many clinical trials during the last decade, and the majority of these were randomized clinical trials (RCTs) that compared the efficacy of oral melatonin with oral midazolam, which was the preferable agent for pediatric premedication in last three decades [10-13]. The MT1 and MT2 receptors are activated by the naturally occurring pituitary hormone melatonin, which possesses hypnotic properties. It has been reported to raise sedative levels without affecting orientation and cause preoperative anxiolytics. A few studies have looked into preoperative oral melatonin (0.2-0.5 mg/kg) in young patients [14]. In this study, 40 patients received melatonin, with a mean age of 6.68 years, a gender ratio of 75.0% to 25.0%, and a mean weight of 22.85 kg. Another 40 patients received a placebo, and this group was similar to the intervention group in terms of age, gender, weight, ASA classification, and underlying cardiac disease. This suggests that the randomization process was successful in achieving a balanced distribution of subjects with varying levels of health and comorbidities. It was observed that there are significant differences between the melatonin and placebo groups in terms of the ease of venipuncture, particularly regarding crying. The M group had a significantly lower proportion of children who cried during venipuncture compared to the P group. This is indicated by the chi-square value of 16.817 and a *P*-value of 0.001, suggesting that the administration of melatonin may have a beneficial effect in reducing the occurrence of crying during venipuncture.

According to the currently available literature, there is no agreement on the ideal melatonin dosage for sedation in children. Melatonin dosage for sedation in children is reported to range between 0.3 and 20 mg in earlier research by Kain et al. [15]. A maximum dosage of melatonin of up to 20 mg has been given to older children without causing any adverse effects other than sedation. Furthermore, the best dosage to cause sedation is still unknown. According to prior research, in this study, greater doses of up to 20 mg may be necessary to reduce anxiety. Larger doses of melatonin were not employed in our trial due to patient safety concerns, although they were recommended in previously published literature. Given that we utilized a low amount of melatonin in our study (0.2 mg/kg), it may be a potential explanation for the greater anxiety levels in that group [15,16].

Patel and Kurdi [16] found that melatonin premedication had sedative effects better than placebo. Logani et al. [17] in their trial on 25 children undergoing dental procedures found that melatonin has a potent sedative effect. Both of the previous trials are in line with our results. In the adult population, the sedative properties of melatonin are still debatable [18]. The sedative effect of melatonin premedication had been proven in the results of Seet et al. [19] in females but not in males. In 2020, in their meta-analysis, Oh et al. suggested that melatonin might have no sufficient evidence for acute postoperative or procedural pain, but it can be used in the treatment of chronic pain [20].

The analgesic effect of intravenous melatonin during the perioperative period was not supported by Andersen et al. [21]. In a placebo study conducted by Laosuwan et al. [22] in 2020, they found that perioperative melatonin had many advantages such as low pain score, lower postoperative fatigue, and good patient satisfaction when compared to placebo in women undergoing elective hysterectomy. Premedication with melatonin was found to be an effective and safe medication, and it provided mild sedation in the preoperative period without any untoward effect in patients undergoing laparoscopic cholecystectomy surgery [23]. 

In a pilot study conducted by Gitto et al. [24] in 2016, they illustrated that melatonin boosts the effectiveness of propofol in pediatric patients as well. Furthermore, when it comes to sedation levels appropriate for children, melatonin proves to be just as efficient as midazolam. These findings justify employing melatonin as a premedication option for pediatric surgical cases [24]. According to the findings of a meta-analysis study conducted by Wang et al. [25], melatonin can decrease postoperative pain scores to a minor degree, reduce postoperative opioid consumption, and decrease the number of patients with analgesic requirements [25]. These results were supported by Tunay et al. [26] when they compared the postoperative analgesic effects of melatonin and vitamin C and found that 6 mg of melatonin can reduce the pain score in the early postoperative period. In 2015, Kirksey et al. [27] did not prove that melatonin has pain-reducing properties in adult patients.

These results matched the results of Samarkandi et al. [28] and Kain et al. [15]. The primary limitations of the study were parental refusal and the relatively small number of cardiac catheterization procedures conducted monthly in pediatric patients with congenital heart defects.

## Conclusions

According to the findings, it can be inferred that melatonin exhibited favorable outcomes across the majority of variables examined in this study. Oral melatonin at a dosage of 0.5 mg/kg seems to be an effective drug for alleviating preoperative anxiety in children. Additionally, the results indicated the considerable efficacy of melatonin in inducing significant sedation and facilitating various procedures for children, notably venipuncture and the application of masks during anesthesia induction.
